# The Amount of Time Dilation for Visual Flickers Corresponds to the Amount of Neural Entrainments Measured by EEG

**DOI:** 10.3389/fncom.2018.00030

**Published:** 2018-05-07

**Authors:** Yuki Hashimoto, Yuko Yotsumoto

**Affiliations:** Department of Life Sciences, University of Tokyo, Tokyo, Japan

**Keywords:** time perception, duration perception, neural entrainment, time, EEG

## Abstract

The neural basis of time perception has long attracted the interests of researchers. Recently, a conceptual model consisting of neural oscillators was proposed and validated by behavioral experiments that measured the dilated duration in perception of a flickering stimulus (Hashimoto and Yotsumoto, 2015). The model proposed that flickering stimuli cause neural entrainment of oscillators, resulting in dilated time perception. In this study, we examined the oscillator-based model of time perception, by collecting electroencephalography (EEG) data during an interval-timing task. Initially, subjects observed a stimulus, either flickering at 10-Hz or constantly illuminated. The subjects then reproduced the duration of the stimulus by pressing a button. As reported in previous studies, the subjects reproduced 1.22 times longer durations for flickering stimuli than for continuously illuminated stimuli. The event-related potential (ERP) during the observation of a flicker oscillated at 10 Hz, reflecting the 10-Hz neural activity phase-locked to the flicker. Importantly, the longer reproduced duration was associated with a larger amplitude of the 10-Hz ERP component during the inter-stimulus interval, as well as during the presentation of the flicker. The correlation between the reproduced duration and the 10-Hz oscillation during the inter-stimulus interval suggested that the flicker-induced neural entrainment affected time dilation. While the 10-Hz flickering stimuli induced phase-locked entrainments at 10 Hz, we also observed event-related desynchronizations of spontaneous neural oscillations in the alpha-frequency range. These could be attributed to the activation of excitatory neurons while observing the flicker stimuli. In addition, neural activity at approximately the alpha frequency increased during the reproduction phase, indicating that flicker-induced neural entrainment persisted even after the offset of the flicker. In summary, our results suggest that the duration perception is mediated by neural oscillations, and that time dilation induced by flickering visual stimuli can be attributed to neural entrainment.

## Introduction

A major focus of interval-timing studies has been the mechanism of how physical time-flow is converted into a mental representation of duration. Many studies have proposed that neural oscillators with periodic activations are utilized for the physical-mental conversion of time, but physiologic aspects of the oscillators are still controversial (Gibbon et al., 1984; Treisman et al., 1994; Matell and Meck, 2004).

In psychophysical studies, flickering visual stimuli have been widely used to investigate the function of the oscillators. It has been reported that a flickering stimulus causes observers to overestimate the duration of the stimulus, and such an overestimation is called “time dilation” (Treisman and Brogan, 1992; Kanai et al., 2006; Ortega and López, 2008). Hashimoto and Yotsumoto (2015) examined time dilation using various flickering frequencies, and conducted simulations by a model that integrated flicker-induced neural entrainments with a previously proposed oscillator-based model (Matell and Meck, 2004). The behavioral results were consistent with the simulations, indicating that neural entrainment can account for flicker-induced time dilation.

The neurophysiological aspect of the flicker-induced time dilation has also been investigated. Herbst et al. (2013) reported that a set of stimuli flickering above the flicker fusion frequency (Landis, 1954) evoked steady-state visually evoked potentials (SSVEPs; Regan, 1977), as the stimuli were not perceived as a flicker and did not cause time dilation. Therefore, they concluded that conscious perception of a flicker, instead of neural activity triggered by a flickering stimulus, played a crucial role in time dilation.

However, EEGs were not recorded in Herbst et al. (2013) during their interval-timing task. Morillon et al. (2009) showed that neural activity differs when attending to the temporal aspect of an event, and when attending to other aspect such as color. They reported larger BOLD activities in dorsolateral prefrontal cortex and temporal-parietal junction when the subjects attended to the temporal aspect, suggesting the temporal processing network is controlled by attention. Therefore, neural activities while attending to the duration of the flicker might be different from neural activities during passive observation of the flicker. To investigate the effect of a flicker on the interval-timing network, it is essential to examine neural activity while subjects attend to the temporal aspect of the flicker stimuli.

In this study, we investigated the physiological relations between neural oscillation and time perception. Recently, we proposed a model which assumed that multiple oscillators with various intrinsic frequencies process interval-timing (Hashimoto and Yotsumoto, 2015). The model extended the striatal beat-frequency model (Buhusi and Meck, 2005) which hypothesized that a duration is encoded as the timing on which a specific subset of oscillatory neurons simultaneously activates. We further simulated the activity of the oscillators when a flicker entrained the oscillators. We demonstrated that when the frequencies of oscillators were drawn to the flickering frequency, the simultaneous activation of the oscillatory neurons occurred earlier than the encoded duration, which in turn caused time dilation. In previous studies of time distortion, entrainment of oscillators was considered a factor capable of inducing time distortion (Treisman and Brogan, 1992; Treisman et al., 1994), while the presentation of flickering stimuli mainly caused time dilation (Treisman and Brogan, 1992; Kanai et al., 2006; Ortega and López, 2008; Kaneko and Murakami, 2009). Hence, neural entrainment was not considered the dominant source of flicker-induced time dilation; instead, changes in arousal level (Treisman and Brogan, 1992; Ortega and López, 2008) and temporal cueing (Kanai et al., 2006; Kaneko and Murakami, 2009; Herbst et al., 2013) became the focus of increased research. Hashimoto and Yotsumoto's model successfully demonstrated time dilation and lack of time contraction by combining neural entrainment with an existing neural model of time perception. In addition, their model can be physiologically verified because it is directly linked to a neural model of time perception and flicker-induced time dilation.

In the present study, we took our model as a working hypothesis, and recorded EEG data while subjects performed a duration reproduction task. First, we measured the EEG power spectrum while subjects observed a flicker. The model predicts that presentation of a flicker would entrain the time-encoding neural network and affect neural oscillations in the brain. Consequently, the neural activities would be phase-locked to the flicker, and the neural activities may last even after the disappearance of the flicker. Second, we examined whether the reproduced duration of a flickering stimulus and the amplitude of the SSVEP would correlate trial by trial. The model predicts that an increase in time dilation would be observed with an increase in neural entrainment, which would be observed as a greater SSVEP.

Additionally, we analyzed the EEG recordings for duration reproduction when no flicker was presented. Some previous studies have reported that the effect of flicker-induced time dilation lasted after the offset of the flicker (Johnston et al., 2006; Burr et al., 2007). In addition, neural entrainment was reported to last ~0.5 s after the offset of the flicker (Spaak et al., 2014). Therefore, we analyzed EEG recordings during the reproduction phase as well as the flicker observation phase.

## Methods

### Subjects

Thirteen volunteers (4 men; age range, 18–23 years) with normal or corrected-to-normal vision participated in the experiment. One subject was excluded from the analyses because Fp1 and Fp2 channel malfunctioned resulting in failure in detecting eye movements and blinks. The data collected from the other 12 subjects (4 men; age range, 18–23 years) were used in the following analyses. All participants were blind to the purpose of the study. This study was carried out in accordance with the recommendations of the ethics boards of the University of Tokyo with written informed consent from all subjects. All subjects gave written informed consent in accordance with the Declaration of Helsinki. The protocol was approved by the institutional review boards of the University of Tokyo.

### Apparatus

The experiment was conducted in a dark soundproof room. The stimuli were presented on a 23.6-inch LCD monitor with a 120-Hz refresh rate, 1,920-pixel width and 1,090-pixel height (VIEWPixx 3D; VPixx Technologies Inc., Saint-Bruno, QC Canada). The viewing distance was set to 57.3 cm with a chin rest. The experiment was conducted with MATLAB 2014 (Mathworks, Natick, MA USA) and the Psychophysics Toolbox extensions (Brainard, 1997; Pelli, 1997; Kleiner et al., 2007).

EEG recordings were obtained at a sampling rate of 512 Hz using a 32-channel EEG system with a signal amplifier, active electrodes, a battery box (g.USBamp, g.LADYbird, and g.GAMMAbox, respectively; g.tec medical engineering, Schiedlberg, Austria) and Simulink with MATLAB 2012. The electrodes were mounted using an AsiaCap (BrainProducts, Gilching, Germany) on the following positions: Fp1, Fp2, AFz, Fz, F3, F4, F7, F8, FC1, FC2, FC5, FC6, T7, T8, Cz, C3, C4, CP1, CP2, CP5, CP6, Pz, P3, P4, P7, P8, POz O1, O2. Additionally, three electrodes were mounted on the left-side of the left-eye, the right-side of the right-eye, and the bottom of the left-eye to monitor for eye movements and blinking. The ground electrode was mounted on Fpz and the reference electrode was mounted on the left earlobe. The ERP and time-frequency representation analyses were conducted with Fieldtrip software (Oostenveld et al., 2011) and custom MATLAB scripts.

### Stimuli

A circular disc with a 4° radius was presented against a black background at the center of the display, and a fixation cross was overlaid on the circular disc. For each subject, the luminance of the circular disc was set to be subjectively equiluminant to 25 cd/m^2^ white by the heterochromatic flicker photometry with a 20-Hz square-wave (Bone and Landrum, 2004) to reduce eyestrain and the effect of luminance adaptation. The luminance of the fixation cross was also set to be subjectively equiluminant to 12.5 cd/m^2^ white.

### Procedure

**Figure 1:**
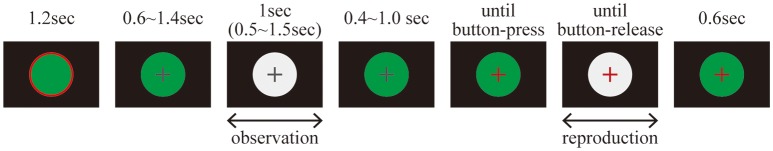
Time course for the duration reproduction task.

EEG data were recorded while the subjects performed a duration reproduction task. The time course of the reproduction task is illustrated in Figure 1.

Before each trial, a green circular disc with a red edge annulus was presented on the display for 1.2 s; during this time the subjects were allowed to blink, but not otherwise. A trial started with a green circular disc overlaid by a gray fixation cross that was presented for 0.6–1.4 s. It was followed by the presentation of a white circular disc, which defined the standard duration that the subjects were asked to remember. The white circular disc was either continuously illuminated or flickering at 10 Hz. After a reproduction cue was delivered by changing the color of the fixation cross from gray to red, the subjects reproduced the remembered standard duration by pressing a space key with a right index finger. The inter-stimulus interval (ISI) between the first presentation of a white circular disc and the reproduction cue was randomly chosen from a range of 0.4–1.0 s. During the reproduction phase, a white circular disc was again presented on the display as visual feedback. As soon as the key was released, the white circular disc disappeared, and a green circular disc replaced it for 0.6 s.

It should be noted that the luminance of the white circular disc was set at the same value for all conditions. Therefore, the maximum luminance was similar for all stimuli, while the temporal average of the luminance was lower for the flickering than for the constantly illuminated stimuli. This setting was a conservative choice in order to avoid false positives of time dilation and SSVEP. If the averaged luminance had been controlled using a brighter flicker, it might have led to an overestimation of time dilation (Xuan et al., 2007) and visually evoked potentials (Norcia et al., 2015), due to brightness. To avoid these critical false positives, we chose to control for maximum luminance and not averaged luminance.

Each subject performed 300 experimental trials and 60 catch trials, resulting in a total of 360 trials. In the experimental trials, the standard duration was always 1.0 s, while in the catch trials the duration of the standard duration was jittered between 0.5 and 1.5 s. There were 150 experimental trials and 30 catch trials in which the first white circular disc was continuously illuminated (“static” condition). In the other 150 experimental trials and 30 catch trials, the first white circular disc was flickering (“flickering” condition). The 360 trials were divided into 30 blocks. The subjects were allowed to take a break anytime between these 12 blocks.

### Behavioral data analysis

To remove trials with extraordinary short or long reproduction, which was caused by instantaneous button press and overlook of the offset signal, the extraordinary reproductions were detected by applying distanced-based outlier detection with ε = 0.1 s and π = 10^−5^ (Knox and Ng, 1998) for each subject and condition, resulting excluding 0.6% of trials from the following analyses.

For each subject, a *t*-test was applied to evaluate the difference in the reproduced durations in the experimental trials between the “static” and “flickering” conditions to determine whether the subjective duration of a flickering stimulus dilated. In addition, for the catch trials in which the standard duration differed trial-by-trial, the correlation between the standard durations and the reproduced durations were tested for each subject and for each condition to confirm that the subjects attended to the duration of the stimuli.

For subsequent EEG analysis, the 150 experimental trials for each stimulus type were sorted in accordance with the subjects' reproduced durations. The top 50 trials were classified as the “long” reproduction trials; the bottom 50, “short”; the intermediate 50, “middle.”

### Electroencephalogram data analysis

#### Preprocessing

An online high-pass filter at 0.1 Hz and an online notch filter at 50 Hz were applied to the EEG data, while recording. The recordings were divided into epochs from the beginning to the end of the trial, followed by an application of an offline band-pass filter from 0.2 to 128 Hz and an offline notch filter. Eye movements and blinks were detected as transient fluctuations in the electrooculogram (EOG), and the trials containing the artifacts were excluded. After the trial removal, independent component analysis (ICA) was applied to the 29 EEG channels without EOGs to correct for muscle artifacts (Makeig et al., 1996). By visual inspection of the independent components for each subject, low-frequency (<1 Hz) components bisymmetrically distributed around frontal electrodes and high-frequency (>20 Hz) components evident only at a few parietotemporal electrodes were excluded as the muscle artifacts originated from the forehead and temple. Subsequently, the EEG was reconstructed by the remaining independent components and the following analyses were applied to the reconstructed EEG (Jung et al., 2000).

#### Event-related potentials

Each trial was divided into two phases. The observation phase was defined as a period of −0.6 to 1.6 s, time-locked to the onset of the standard duration; and the reproduction phase was defined as a period of −0.84 to 1.14 s, time-locked to the onset of reproduction. For each subject and stimulus type, the ERP was calculated by averaging the preprocessed signals. For each ERP, baseline correction was applied by subtracting the mean signal within an interval of −0.1 to 0 s. Then, the ERPs acquired from Pz, P3, P4, POz, O1, and O2 were averaged.

For each of the 1-s standard duration intervals and the 0.4-s interval occurring just after the offset of the standard duration, the SSVEP amplitude was calculated by applying the discrete Fourier transform (Cooley et al., 1969) to the ERP for the intervals measured for each subject. The detailed formulation is provided as Supplementary Formula 1. For each of the intervals, a within-subject *t*-test was applied to evaluate the differences in the SSVEP amplitude between “static” and “flickering” conditions. The amplitudes of the second (20 Hz), third (30 Hz), and fourth (40 Hz) harmonics were also compared for the “static” and “flickering” conditions using within-subject *t*-tests.

To further investigate the relationship between behavior and SSVEP, the SSVEP amplitudes of the “long,” “middle,” and “short” reproduction trials were modeled and evaluated using analysis of variance (ANOVA) followed by a *post-hoc* Tukey's honest significant difference (HSD) test. The type of reproduced duration (“long,” “middle,” or “short”) was set as a fixed effect and the subject was set as a random effect in the model. Cohen's *d*s (Cohen, 1988) was calculated for each difference (Equation 1).

(1) $$Cohen's\;d=\frac{sample\;mean}{sample\;standard\;deviation}\times \sqrt{2}$$

In addition, for each of the second, third, and fourth SSVEP harmonic values, the differences in the respective amplitudes of the “long,” “middle,” and “short” reproduction trials were tested by ANOVA and the *post-hoc* Tukey's HSD test. The fixed and random effects were set as those for the ANOVA for the base frequency. Cohen's *d*s values were also calculated in the same manner.

#### Time-frequency representations

The time-frequency representation was calculated for each trial by projecting the preprocessed signal onto the time-frequency representation using a short-term Fourier transform and applying an adaptive Hanning window length. The window length of the Hanning taper was set at 7 cycles per window. For each time-frequency representation, a baseline-correction was applied by subtracting the mean amplitude within an interval of −0.1 to 0 s for each frequency. Following the baseline-correction, the time-frequency representations were averaged for each subject and stimulus type and subsequently, the time-frequency representations acquired from Pz, P3, P4, POz, O1, and O2 were averaged.

The difference in time-frequency representation between the “static” and “flickering” conditions was tested by a cluster-based permutation (Maris and Oostenveld, 2007) with *ft_timelockstatistics* function in the Fieldtrip software. In the cluster-based permutation test, two conditions were compared by calculating *t*-values for every time-frequency data point. A continuum in which the *t*-value exceeded a certain criterion was clustered and *t*-values in the cluster were summed up, resulting in a *T*-value of the cluster. If there were multiple *T*-values originating from multiple clusters, *T*-values, except the largest one, were rejected. To compute the distribution of *T*-values based on the null hypothesis, the label of condition was randomly assigned to the data sets, and *T*-values were resampled repeatedly. The position of the original *T*-value and the resampled *T*-values were sorted and the percentile of the original *T*-value was calculated. If the percentile of original *T*-value was smaller than 2.5% or larger than 97.5%, the two conditions were concluded to be significantly different.

## Results

### Behavior

The mean reproduced duration for the “static” condition was 0.95 s (SD: 0.08 s) and 1.16 s (SD: 0.19 s) for the “flickering” condition. The difference was significant for all subjects (*p* < 1.0 × 10^−5^ for each subject, *p* < 5.0 × 10^−4^ with Bonferroni correction), which indicated that the flicker was perceived to be longer than the constantly illuminated stimulus. The correlation between the standard duration and the reproduced duration in catch trials was 0.78 (SD: 0.11) for the “static” condition, and 0.76 (SD: 0.09) for the “flickering” condition. The correlation was significant in all subjects and conditions (*p* < 1.0 × 10^−3^ for all subjects and conditions, *p* < 0.05 with Bonferroni correction), which indicated that the subjects attended to each standard duration accurately. Additional information regarding the distribution of the reproduced duration for each subject and condition is provided in Supplementary Figure 1, and the detailed results of the correlation analyses are reported in Supplementary Table 1. The reproduced durations in the “long,” “middle,” and “short” reproduction trials in the “static” and “flickering” conditions were shown in Table 1. The mean differences of reproduced duration between the “long” and “short” reproduction trials were 0.24 s (SD: 0.06 s) and 0.30 s (SD: 0.10 s) in the “static” and “flickering” conditions, respectively.

**Table 1:** Mean and SD of reproduced duration of the trials categorized as “long,” “middle,” and “short.”

		**“Long”**	**“Middle”**	**“Short”**
Constantly illuminated	Mean	1.07	0.95	0.83
	(S.D.)	(0.09)	(0.08)	(0.09)
Flickering	Mean	1.31	1.15	1.00
	(S.D.)	(0.22)	(0.19)	(0.18)

### Electroencephalogram

#### Steady state visually evoked potential

**Figure 2:**
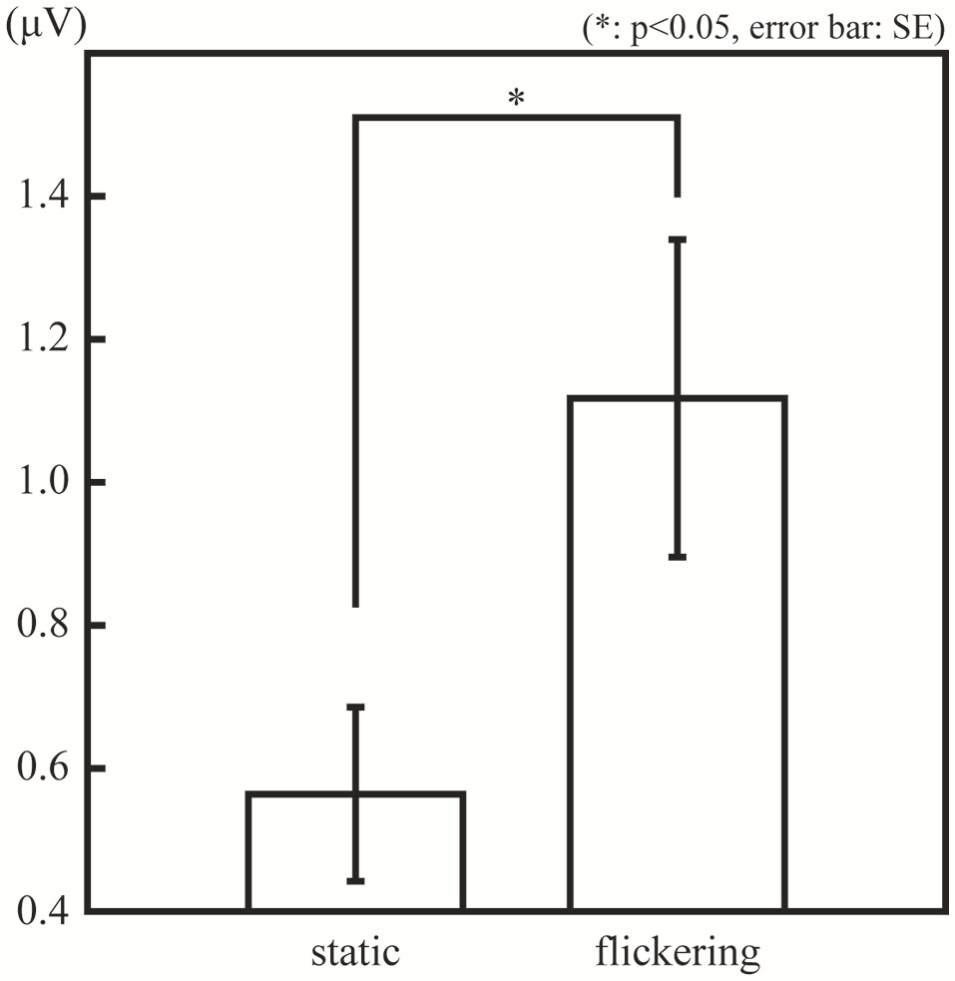
The amplitude of the 10-Hz ERP components in the observation phase in the “static” and “flickering” conditions. The asterisk indicates the significant difference with α = 0.05. The error bars represent the standard errors of the mean.

Figure 2 illustrates the amplitude of the SSVEP (10-Hz ERP component) in the observation phase. The amplitude of the SSVEP was significantly larger in the “flickering” condition than in the “static” condition [*t*_(11)_ = 3.02, *p* = 0.01], suggesting the flickering stimulus evoked a 10-Hz neural activity phase-locked to the change in luminance of the flicker. The amplitudes of the second, third, and forth SSVEP harmonics were also significantly larger in the “flickering” condition than in the “static” condition [*t*_(11)_ = 6.36, 2.41, 3.85; *p* = 0.0001, 0.03, 0.002, respectively]. The topographic representation and the frequency spectrum of the SSVEP are illustrated in Supplementary Figures 2,3, respectively.

**Figure 3:**
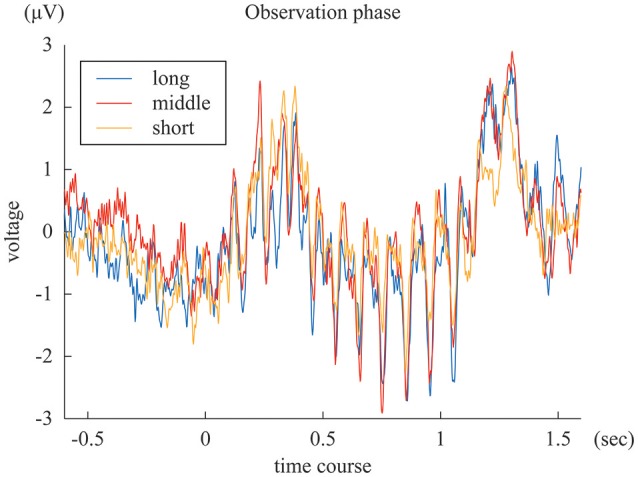
The blue, red, and yellow lines represent ERP of “long,” “middle,” and “short” reproduction trials, averaged across subjects.

**Figure 4:**
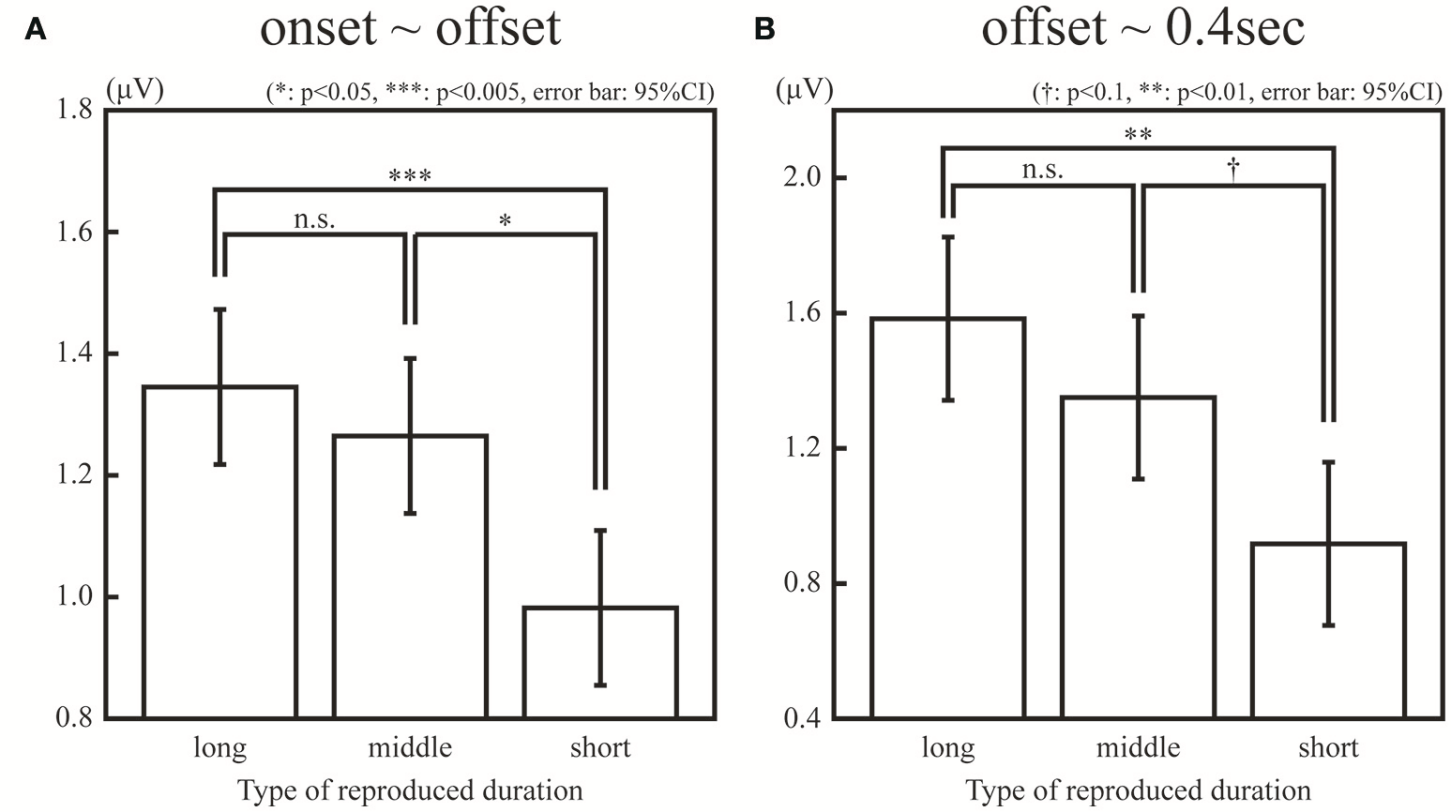
Amplitudes of the 10-Hz ERP components in the “long,” “middle,” “short” reproduction trials during observation of a flicker **(A)** and after the offset of the flicker **(B)**. The single, double, and triple asterisk(s) indicate the significant difference with α = 0.05, 0.01, and 0.005, respectively. The dagger (†) indicates the marginally significant difference with α = 0.1.

The SSVEPs during the 1-s standard duration in the “long”, “middle”, and “short” reproduction trials were illustrated in Figure 3. The amplitudes of the SSVEP in the “long,” “middle,” and “short” reproduction trials were 1.35 μV (SD: 0.89 μV), 1.26 μV (SD: 0.81 μV), and 0.98 μV (SD: 0.66 μV), which are illustrated in Figure 4A. ANOVA revealed that the amplitudes of the SSVEP were different across the types of reproduced duration [*F*_(2, 22)_ = 8.20, *p* = 0.004 with and without Greenhouse-Geisser's sphericity correction, ε = 0.98]. The *post-hoc* Tukey's HSD test showed significant differences in the amplitude of the SSVEP between the “long” and “short” reproduction trials, and between the “middle” and “short” reproduction trials (*p* < 0.005 and *p* < 0.05, Cohen's *d* = 1.26 and *d* = 1.53, respectively). There was no significant difference in the SSVEP between the “long” and “middle” reproduction trials.

In addition, the SSVEPs during the 0.4 s immediately after the offset of the standard duration were calculated for each subject and stimulus type. Because this 0.4-s interval was an inter-stimulus interval and the same green circular disc was continuously presented both in the “static” and “flickering” conditions, the difference of the SSVEPs in this interval was not due to the difference of the presented stimuli, but it reflected the difference of the neural entrainment lasting even after the offset of the stimuli. The SSVEP after the offset of the standard duration was significantly larger in the “flickering” condition than in the “static” condition [*t*_(11)_ = 2.46, *p* < 0.05]. The amplitudes in the “long,” “middle,” and “short” reproduction trials in the “flickering” condition were 1.58 μV (SD: 1.44 μV), 1.35 μV (SD: 0.98 μV), and 0.92 μV (SD: 0.72 μV) respectively, which are illustrated in Figure 4B. ANOVA revealed that amplitudes were different across the types of reproduced durations [*F*_(2, 22)_ = 6.18, *p* = 0.007 and 0.02 with and without Greenhouse-Geisser's sphericity correction, ε = 0.67]. The Tukey's HSD test showed a significant difference in the amplitude between the “long” and “short” reproduction trials (*p* < 0.01, Cohen's *d* = 1.09). There was a marginally significant difference between the “middle” and the “short” reproduction trials (*p* < 0.1, Cohen's *d* = 1.34). There was no significant difference between the “long” and “middle” reproduction trials.

The second, third, and forth harmonics of the SSVEP during the standard duration were not significantly different among the “long”, “middle,” and “short” reproduction trials [*F*_(2, 22)_ = 1.82, 0.14, 0.91, respectively; *p* > 0.1 for all harmonics]. The amplitude of the SSVEP harmonics during 0.4 s just after the offset were not significantly different either [*F*_(2, 22)_ = 0.35, 0.70, 0.51, respectively; *p* > 0.1 for all harmonics].

#### Event-related potential and time-frequency representation

**Figure 5:**
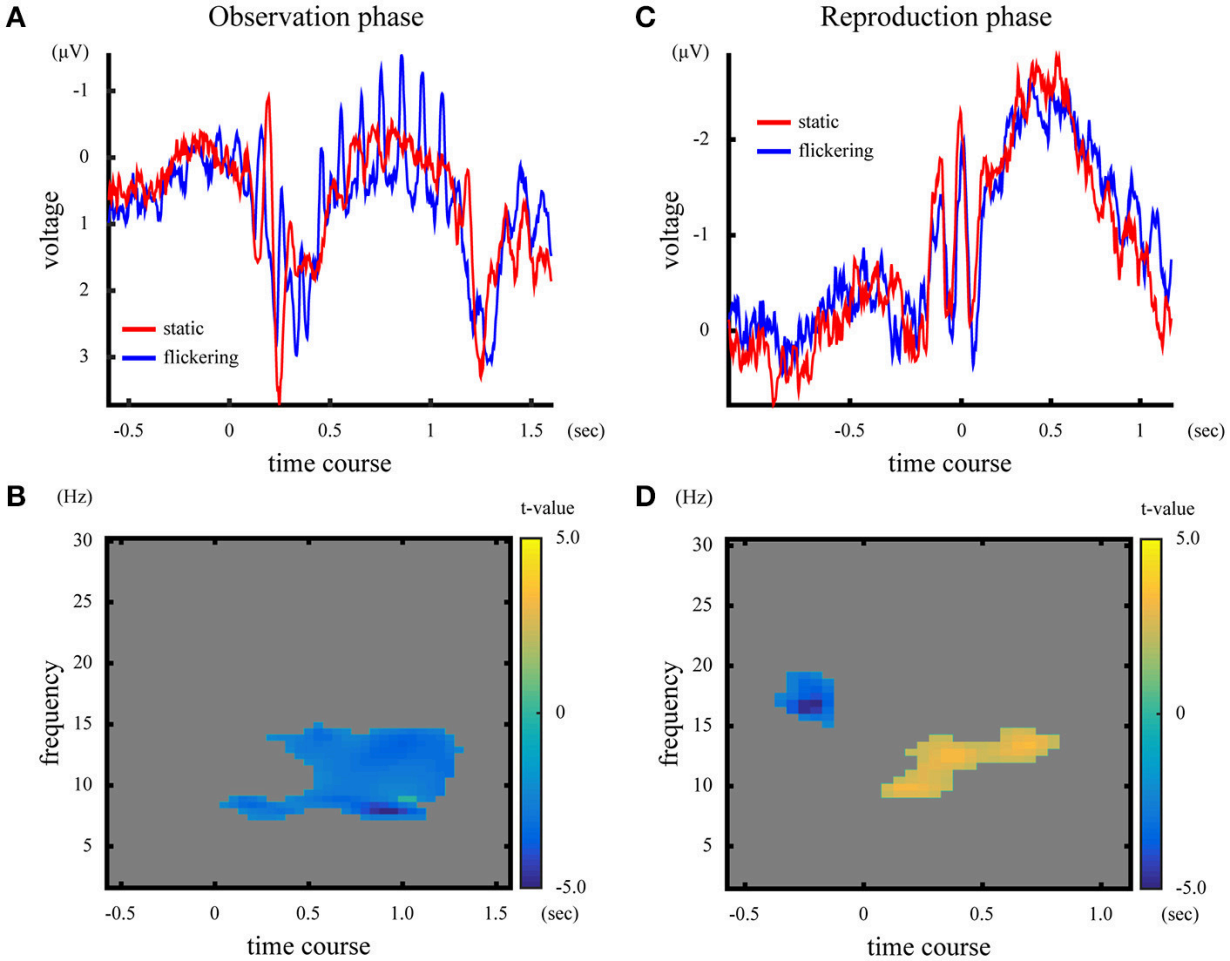
[Upper panels] ERP and time-frequency representation in the observation phase **(A)** and the reproduction phase **(C)**. The blue and red lines represent ERPs in the “static” and “flickering” condition, respectively. [Lower panels] Difference of time-frequency representation between the “static” and “flickering” condition in the observation phase **(B)**, and in the reproduction phase **(D)**. The yellow area indicates that the time-frequency amplitude was larger in the “flickering” condition, while the blue area shows that the time-frequency amplitude was larger in the “static” condition. The gray area indicates no significant differences between the two conditions. The color bar indicates the *t*-value at the time-frequency data point.

The ERPs in the observation phase were averaged across subjects, and shown in Figure 5A. In the observation phase, the 10-Hz flicker caused oscillatory EEG fluctuations at 10 Hz (SSVEP). Figure 5B illustrates the difference in time-frequency representation in the observation phase between the “static” and “flickering” condition, represented by the *t*-values calculated across subjects. The presentation of a flicker decreased the EEG amplitude in the wide range of frequency around 10 Hz (*p* = 0.010), suggesting large event-related desynchronization (Klimesch et al., 2007) at approximately the alpha band in the “flickering” condition.

The averaged ERP and the *t*-values in time-frequency representation in the reproduction phase are illustrated in Figures 5C,D. In the “flickering” condition, the EEG amplitude at approximately the alpha band during the reproduction increased, while before the onset of reproduction the amplitude at approximately the beta band decreased (*p* = 0.018 for the alpha band activity, *p* = 0.048 for the beta band activity). Note that the visual stimuli presented during the reproduction phase did not flicker even in the flickering condition. Additionally, the time-frequency representations of EEG averaged across subjects for each condition and phase are shown in Supplementary Figure 4.

## Discussion

In the present study, we measured the neural correlations between EEG and time dilation in order to evaluate the effects of neural entrainment in time dilation. We found that 10-Hz flickers induced time dilation replicating the results of previous studies, and that the reproduced duration correlated with the amplitude of the 10-Hz ERP component both before and after the flicker was offset.

Flicker-induced oscillations that lasted even after the disappearance of the flicker were also reported by the previous study (Spaak et al., 2014), and the EEG oscillations were considered to reflect neural activity being entrained to the flicker. Therefore, the correlation between the reproduced duration and the amplitude of the flicker-induced 10-Hz oscillation supported our hypothesis that the presentation of a flicker can induce neural entrainment, and that this neural entrainment would cause flicker-induced time dilation. It should be mentioned that a significant difference in the 10-Hz ERP amplitude was not observed comparing long and middle reproduction trials. However, the lack of difference observed in the 10-Hz ERP amplitude does not necessarily suggest a lack of difference in the magnitude of neural entrainment. Rather, it might be more plausible to attribute this lack of difference to the relationship between neural entrainment and the consequent ERP. Because the EEG is the summation of neural activities, it is natural to assume that the oscillatory 10-Hz ERP observed in our results represented the collective activity of the entrained neural oscillators. Mathematically, the phase coherence among oscillators and the amplitude of their mean activity are associated by an S-shaped function (Supplementary Figure 5), thus when comparing two conditions having a stronger neural entrainment, the difference in the ERP amplitude gradually decreases and is less detectable. This property may explain why there was no difference observed in the 10-Hz ERP amplitude comparing the middle and long reproduction trials. Conversely, it is less likely that there was no change in ERP because there was no difference in the magnitude of neural entrainment. Had the magnitude of the neural entrainment been small in the short reproduction trials, and large but identical in the middle and long reproduction trials, the distribution of the reproduced duration would have been skewed. However, such skewness was not observed in our results (Supplementary Figure 1). Therefore, the lack of difference in the ERP amplitude should be attributed to the relationship between the neural entrainment and the resulting ERP, rather than on an similar neural entrainment.

The ERP amplitudes of the harmonics were also larger for the flickering stimuli than for the continuously illuminated stimuli. However, the amplitudes of the harmonics did not correlate with the reproduced duration. The difference might be attributed to the distribution of oscillators in the time-encoding network. In the model proposed in Hashimoto and Yotsumoto (2015), the oscillating frequencies of the time-encoding network distribute more densely at around alpha frequencies compared to other frequencies. Therefore, the entrainment of the oscillators at around 10 Hz would have larger impact on time perception than the entrainment of the other oscillators. The difference of impact might have led to the result that the amplitude of the 10-HZ ERP component correlated with the amount of time dilation while the amplitude of the harmonics did not.

Although the ERP analysis revealed the flicker induced phase-locked 10-Hz neural activity during the observation of a flicker (Figure 4A), decrease of EEG amplitude at ~10 Hz was observed through time-frequency representation (Figure 4B). The decrement of neural activity at ~10 Hz could be attributed to the large amplitude of event-related desynchronization. Klimesch et al. (2007) reviewed that EEG-components at approximately the alpha band often decrease while performing cognitive tasks, and such a decrement is considered to reflect a decrease in spontaneous alpha activity caused by excitatory brain processing. In our temporal reproduction task, the subjects' visual system had to process a greater amount of change in luminance when the stimulus flickered, which could result in higher event-related desynchronization. If the event-related desynchronization exceeded the amplitude of 10-Hz neural activity evoked by the presentation of a flicker, there would have been no cluster exhibiting increased amplitude at 10 Hz with the flickering stimulus. In fact, the 10-Hz neural activation was not evident during the visual inspections of the trial-by-trial EEG analysis. Conversely, in the ERP analysis, averaging canceled out spontaneous neural activities that had randomly distributed phases. Therefore, the 10-Hz SSVEP phase locked to the flicker was clearly observable.

Interestingly, the presentation of a flicker also evoked neural activity at approximately the alpha band, even in the reproduction phase when no flicker was presented. There are some possible explanations for this phenomenon: First, this oscillation might be attributed to the “replay of the flicker in the mind” phenomenon, as in this phase, the subjects remembered the standard duration defined by the presentation of a flicker. However, this explanation is unlikely because the frequency of increased activity was slightly higher than 10 Hz. If the EEG oscillation was because of the “replay of the flicker in the mind” phenomenon, the reproduced duration would be <1.0 s because the higher EEG frequency would indicate subjectively faster elapse of time in the reproduction phase than that in the observation phase. This was not the case with the findings of our behavioral tests. The second possibility is the aftereffect of the neural activation induced by observation of a flicker. Previously, studies have reported that presentation of a flicker altered perception of the subsequent stimulus. Johnston et al. (2006) reported that presentation of a flicker compressed the perceived duration of the subsequent stimulus presented 0.5 s later, suggesting an aftereffect of a flicker on interval-timing. Droit-Volet and Wearden (2002) conducted an experiment with children, and similarly reported the effect of a flicker on the duration perception of the subsequent stimulus. In line with these studies, the increased activity during the reproduction phase could be interpreted as an aftereffect of the neural activation induced by the preceding flicker. This explanation is congruent with the model of neural entrainment. Alpha-band neural entrainment induced by the presentation of a flicker have been reported to last around 0.5 s (Spaak et al., 2014), and in our results, the sustained neural entrainment also sustained for 0.4 s after the offset of the flicker. Therefore, the alpha oscillatory EEG data observed in our study might reflect the aftereffect.

In our experiments, we evaluated the correlation between time dilation and neural entrainment by conducting both inter-stimulus comparisons (“static” and “flickering” conditions) and intra-stimulus comparisons (“long” and “short” reproduction trials). The results supported the hypothesis that neural entrainment induces subjective time dilation. However, in our studies, we only measured EEGs having continuously illuminated stimuli and 10-Hz flickers. It would be of value to conduct additional experiments with stimuli flickering at different frequencies in order to better examine whether the correlation between neural entrainment and time dilation is a general phenomenon. In addition, recording EEGs with arrhythmic flickers will also be helpful in distinguishing the neural entrainment due to oscillators, the neural activities reflecting each flash in the flicker and, in particular, it will contribute to identify the ultimate source of event-related desynchronization reflecting excitatory brain processing induced by the flicker. Despite these reservations, our results clearly showed that the observation of a flicker during an interval-timing task evoked a periodic neural activity, which persisted even after the offset of the flicker, and the prolonged perception was associated with a larger periodic neural activity.

In summary, (1) presentation of a flicker induced subjective time dilation, and the amount of time dilation correlated with the amplitude of neural entrainment induced by the flicker. (2) The observation of a flicker evoked large event-related desynchronization at approximately the alpha band, suggesting excitatory brain processing. (3) An aftereffect of the flicker was observed during the reproduction phase because of the increase in EEG amplitude at approximately the alpha band. These results indicate that neural entrainment can be triggered by the presentation of a flicker, and support the working hypothesis that neural entrainment results in the distortion of interval-timing perception.

## Author contributions

YH and YY conceived and designed the experiments; YH performed the experiments and analyzed the data; YH and YY wrote the manuscript.

### Conflict of interest statement

The authors declare that the research was conducted in the absence of any commercial or financial relationships that could be construed as a potential conflict of interest.
