# Supplementary material for: The Amount of Time Dilation for Visual Flickers Corresponds to the Amount of Neural Entrainments Measured by EEG
Source: Front Comput Neurosci. 2018 May 7;12:30. doi: 10.3389/fncom.2018.00030 (PMC5949346; doi:10.3389/fncom.2018.00030)
Supplement: Supplementary file 1 [file Data_Sheet_1.docx]

Supplementary Material

The amount of time dilation for visual flickers corresponds to the amount of neural entrainments measured by EEG

Yuki Hashimoto*, Yuko Yotsumoto

*** Correspondence:** Corresponding Author: hashimoto@fechner.c.u-tokyo.ac.jp

# Supplementary Data

**
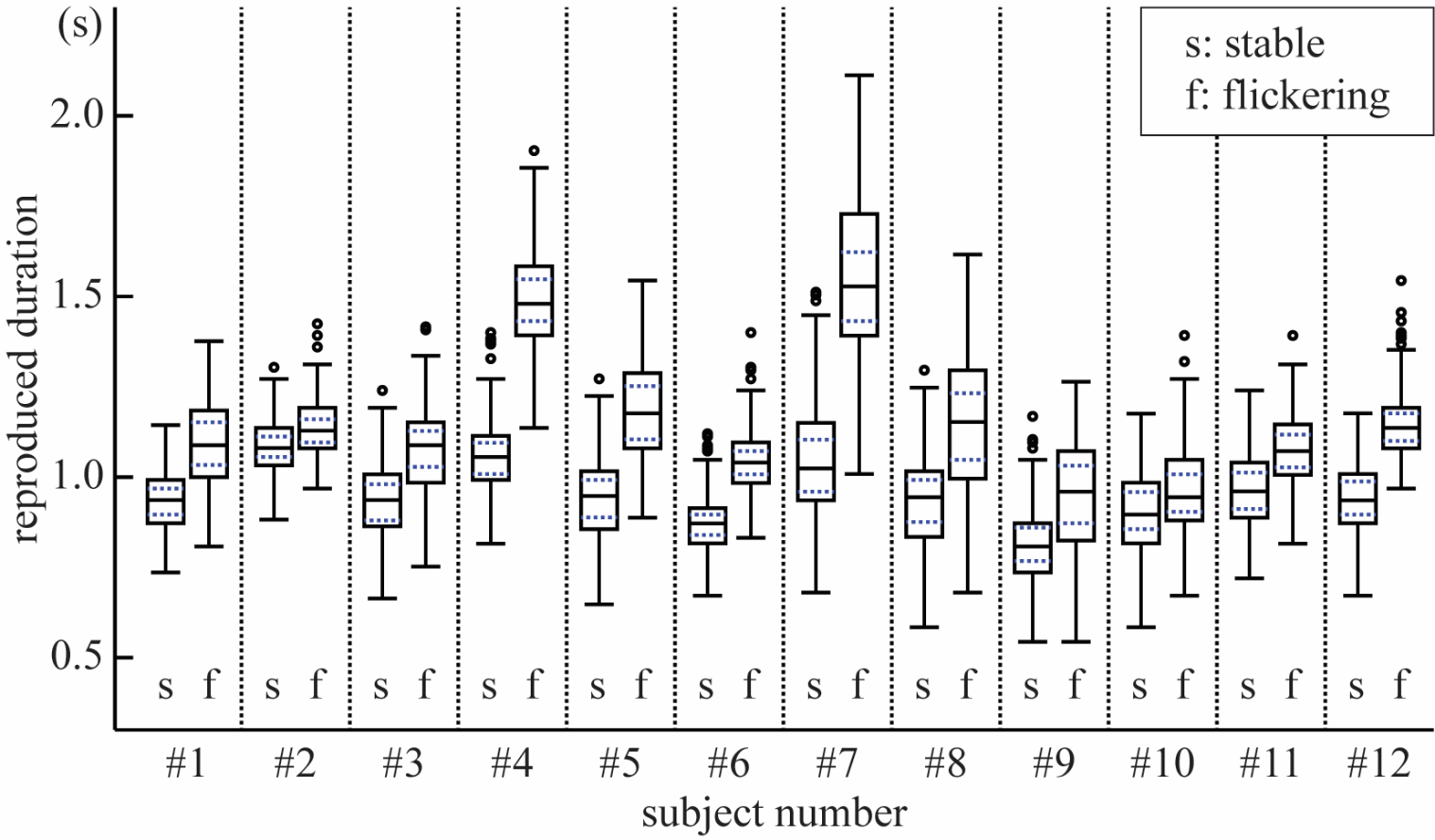
**

**Supplementary Figure 1.** The reproduced durations for each subject and condition. Each column separated by dashed lines represents the data from each subject. The left and right box plots in each column represent the reproduced durations in the “static” and “flickering” condition respectively. The lower and upper limits of the box represent the 1^st^ and 3^rd^ quantiles. The lower and upper limits of the whisker represent the minimum and maximum reproduced durations excluding the outliers. The circles represent the outliers defined as the data points without [Q_1/4_ - 1.5IQR, Q_3/4_ + 1.5IQR] where Q_1/4_ and Q_3/4_ represent 1^st^ and 3^rd^ quantiles, and IQR is defined as the difference between Q_1/4_ and Q_3/4_. The solid line and the blue dashed lines inside the box represent the median and the tertiles.


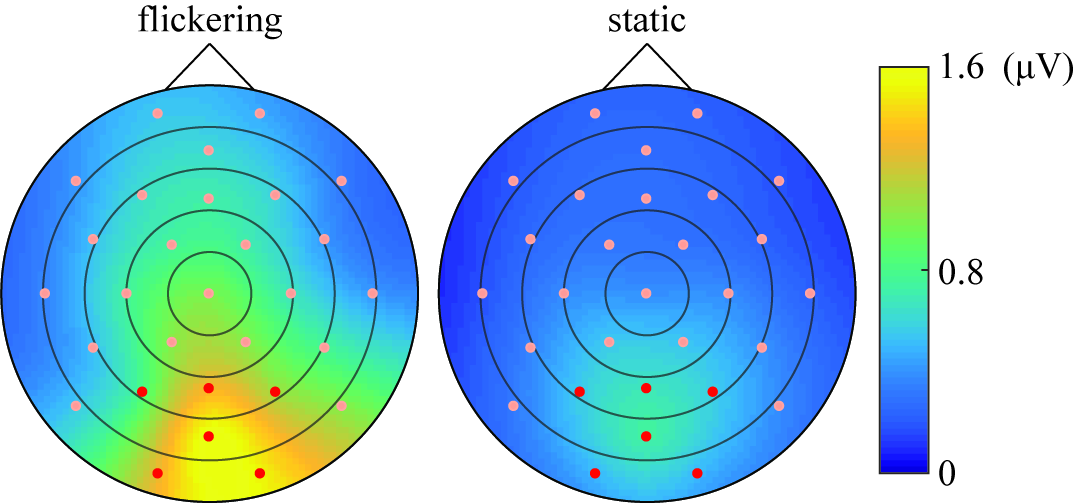


**Supplementary Figure 2.** Conventional topographic representation of the 10-Hz ERP component. For the topography reconstruction, we (1) calculated the ERP of each electrode and subject by averaging preprocessed signals across trials, (2) computed the 10-Hz amplitude of each ERP, (3) averaged the 10-Hz amplitudes across subjects, and (4) applied the (spatial) biharmonic spline interpolation (MATLAB® 4 griddata method) to the averaged 10-Hz amplitudes. The red and pink dots represent the electrodes used and not used in the SSVEP analyses.


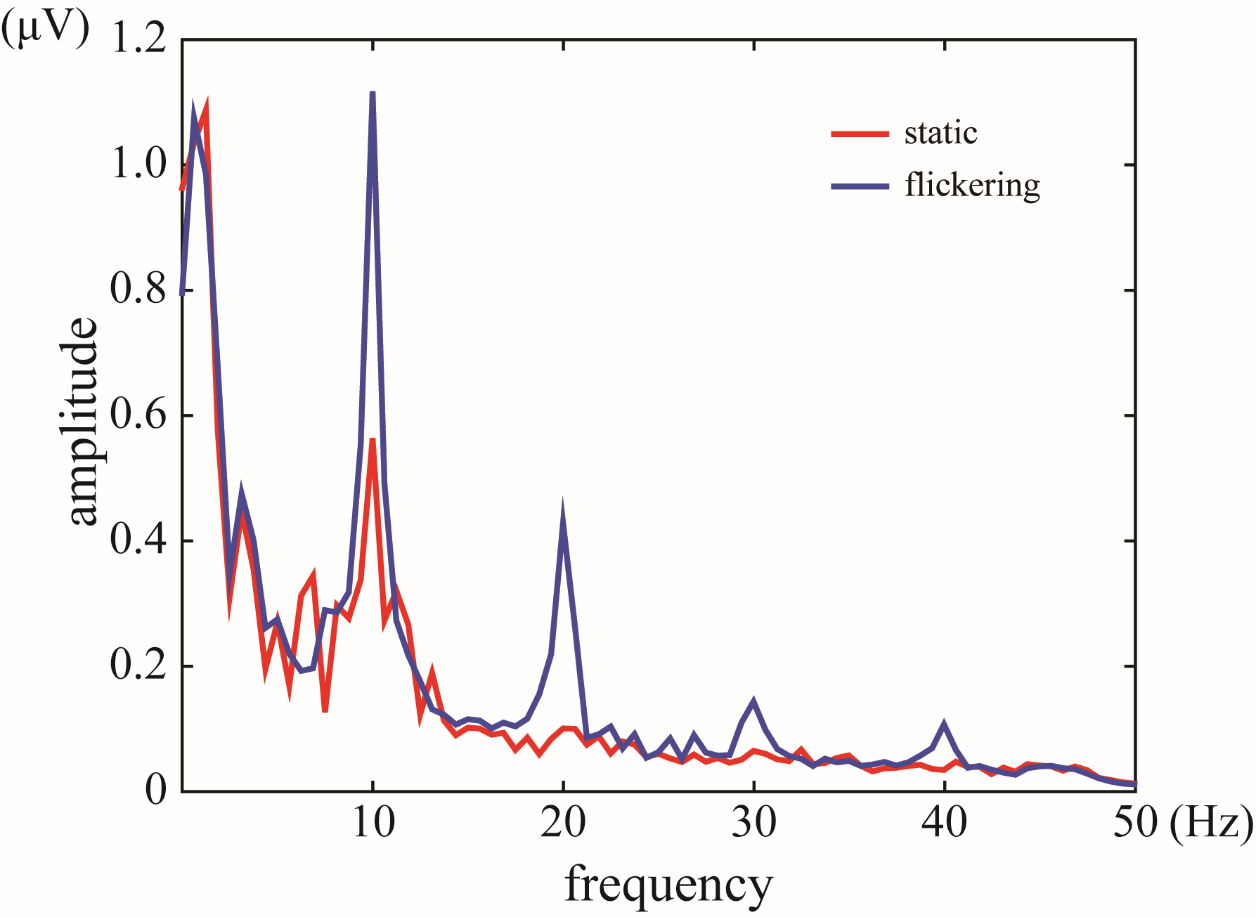


**Supplementary Figure 3.** Frequency spectra of the ERP during the standard duration. The red and lines represent the ERP frequency spectra in the “static” and “flickering” conditions, averaged across subjects.


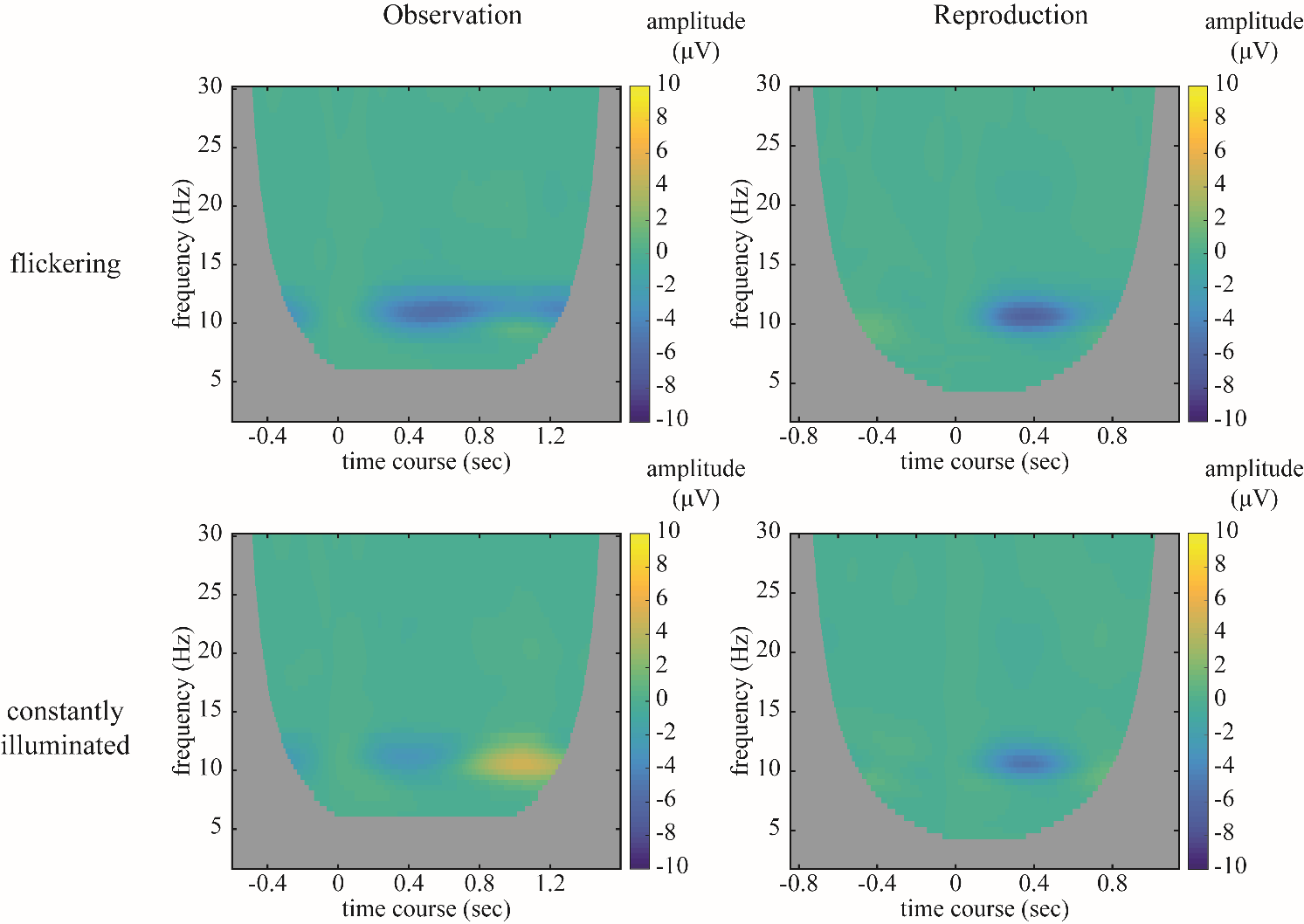


**Supplementary Figure 4.** The time-frequency representations of EEGs during the observation of standard duration and reproduction which are baseline-corrected by subtracting the average amplitude within the interval between -0.1 sec and 0 sec for each frequency bin. The left and right columns show the time-frequency representations during the observation and reproduction, respectively, and the upper and lower rows show the data in the conditions with flickering and constantly illuminated stimuli, respectively.


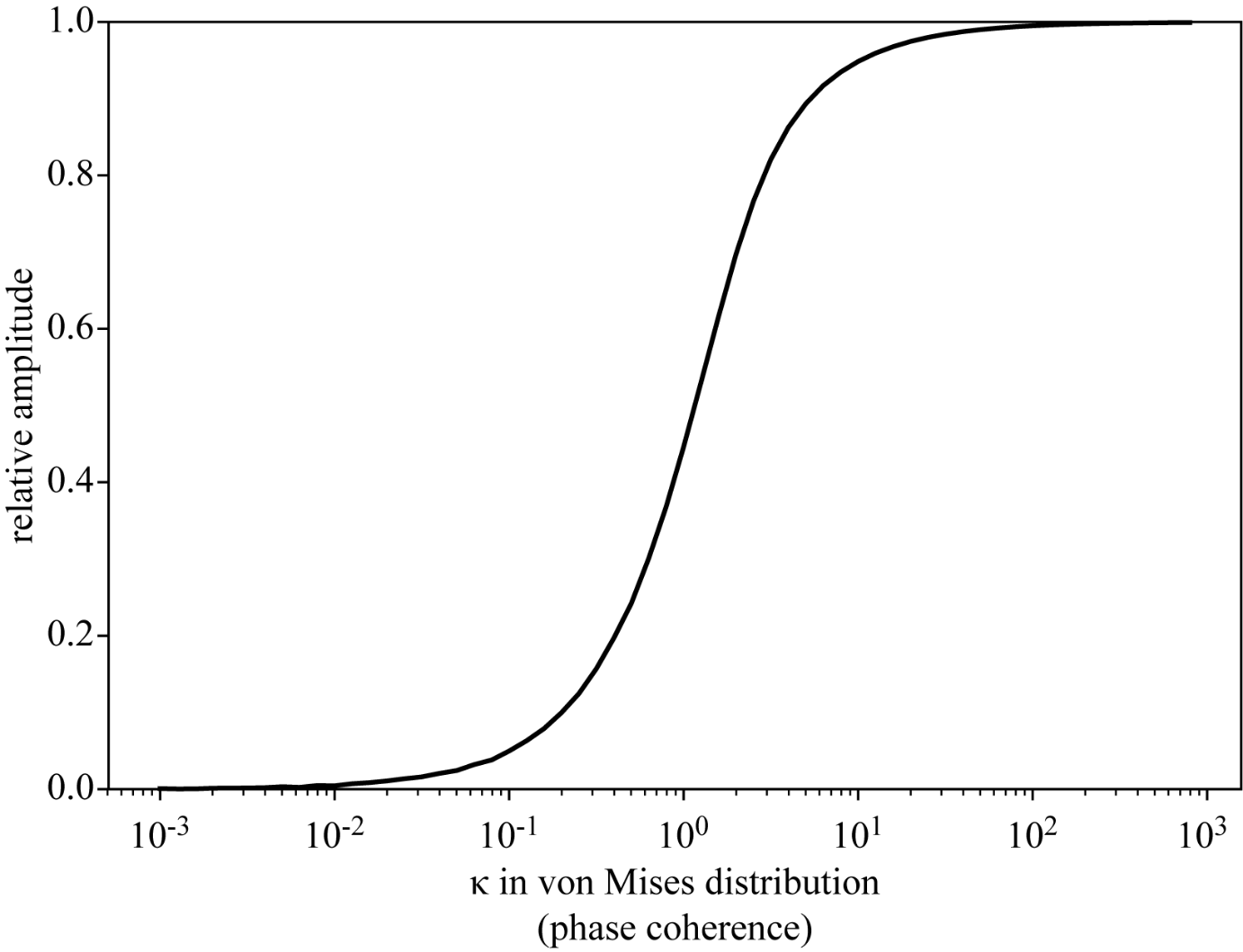


**Supplementary Figure 5.** The relative amplitude of infinite sum of sinusoidal waves whose phase follows von Mises distribution. The $\kappa$ is a parameter of von Mises distribution which represents cohesiveness of the sample angular. The literal formulation was given below.

$$relative\_amplitude\left( \kappa\right)=\lim_{N\to\infty} \frac{\sum_{i=1}^{N} \cos\left( \theta_{i} \right)}{N} \left( \theta_{i}\sim von Mises(0, \kappa) \right)$$

**Supplementary Formula 1.** 10-Hz amplitude was calculated by Equation (1). N represents the number of data points in the analyzed interval. F_s_ represents the sampling frequency, which was 512 in our experiment. Equation (2) is the standard formulation of the discrete Fourier transform.

|  | $10 Hz amplitude=\frac{{2\left\Vert F\left( \frac{10N}{F_{s}} \right) \right\Vert}_{2}}{N}$ | (1) |
| --- | --- | --- |
|  | $F\left( t \right)=\sum_{x=0}^{N-1} f(x)e^{-i\frac{2\pi tx}{N}}$ | (2) |

**Supplementary Table 1.**

| The coefficients in the linear regression of the catch trials data. (reproduced duration ~ 1 + standard duration) | | | |
| --- | --- | --- | --- |
|  | | Constantly illuminated | Flickering |
| Intercept | Mean | 0.27 | 0.40 |
|  | (S.D.) | (0.15) | (0.23) |
| Slope | Mean | 0.64 | 0.73 |
|  | (S.D.) | (0.18) | (0.19) |
